# Testing Ecological Theories of Offender Spatial Decision Making Using a Discrete Choice Model

**DOI:** 10.1177/0011128714540276

**Published:** 2015-04

**Authors:** Shane D. Johnson, Lucia Summers

**Affiliations:** 1 University College London, UK; 2 Texas State University, San Marcos, USA

**Keywords:** theft from motor vehicle, crime location choice, journey to crime, crime pattern theory, social cohesion

## Abstract

Research demonstrates that crime is spatially concentrated. However, most research relies on information about where crimes occur, without reference to where offenders reside. This study examines how the characteristics of neighborhoods and their proximity to offender home locations affect offender spatial decision making. Using a discrete choice model and data for detected incidents of *theft from vehicles (TFV)*, we test predictions from two theoretical perspectives—crime pattern and social disorganization theories. We demonstrate that offenders favor areas that are low in social cohesion and closer to their home, or other age-related activity nodes. For adult offenders, choices also appear to be influenced by how accessible a neighborhood is via the street network. The implications for criminological theory and crime prevention are discussed.

## Introduction

Research demonstrates that the spatial distribution of crime is far from uniform (for reviews, see Eck & Weisburd, 1995; Weisburd, Bernasco, & Bruinsma, 2009) and that crime density reflects more than simple variation in target density. Consequently, a number of theories have been proposed to explain observed patterns of offender spatial decision making. However, much of the associated empirical research relies on the analysis of where crimes occur, without reference to where offenders reside. In the present article, we examine spatial patterns of a high-volume acquisitive crime that has received little attention in the academic literature—*theft from vehicle (TVF)*—and do so using a discrete spatial choice approach (McFadden, 1973). The use of this approach allows us to compare the characteristics of those locations where a sample of offenders chose to commit offenses with those locations that could have been chosen, but were not. In particular, drawing on ecological theories of crime, we consider how and why the composition of urban environments might shape offender targeting decisions, paying particular attention to how the connectivity of areas and the locations of routine activity nodes might affect the likelihood of offenders targeting them. In what follows, we review relevant theoretical perspectives and discuss existing research that has examined offender mobility. We then derive a set of expectations regarding offender spatial decision making and test these using data for a sample of offenders. The article closes with a discussion of the implications of the findings for criminological understanding and policy.

### Background

Scholars have been concerned with the spatial analysis of crime for some time, and have shown it to be spatially clustered at spatial scales ranging from provinces (e.g., Quetelet, 1842/1973), to zones within cities (e.g., Shaw & McKay, 1942), to street segments (e.g., Groff, Weisburd, & Yang, 2010; Johnson & Bowers, 2010; Weisburd et al., 2004), to intersections (e.g., Sherman, Gartin, & Buerger, 1989), to individual locations such as homes (e.g., Pease, 1998), or facilities (e.g., Eck, Clarke, & Guerette, 2007). Moreover, researchers have shown this to be the case for a range of crime types including street robbery (e.g., Alford, 1996; Sherman et al., 1989), burglary (e.g., Brantingham & Brantingham, 1975; Johnson & Bowers, 2010), drug dealing (e.g., Eck, 1994; Rengert, 1996; Rengert et al., 2000), and violent crimes (e.g., Braga, Papachristos, & Hureau, 2010; Bullock, 1955; Davidson, 1989; Morenoff, Sampson, & Raudenbush, 2001; Nelson, Bromley, & Thomas, 2001; Roman, 2005; Sherman et al., 1989; van Wilsem, 2009; Ye & Wu, 2011).

Attempts at explanation vary and consider a variety of mechanisms that might influence the likelihood of crime occurrence at a particular location. In the present study, we consider two different (but compatible) theoretical perspectives: crime pattern and social disorganization theory. In general terms, the two perspectives may be thought of as focusing on how and why an offender may become aware of, and target some locations but not others, and how the social fabric of an area might affect the likelihood of crime occurring within it. We discuss each of these perspectives below but, before doing so, the type of crime to be considered in this article is introduced.

While much research has considered spatial patterns of acquisitive crimes such as burglary and robbery, little research has considered patterns for other types of crime, such as TFV. In the United Kingdom at least, this is a high-volume crime (see Chaplin, Flatley, & Smith, 2011) and hence of interest to scholars and the police alike. While the crime shares characteristics with residential burglary, it is also quite different. For instance, residential burglary is generally a crime of stealth usually committed inside an unoccupied dwelling, out of the sight of those who might intervene, or report the offense to the authorities. In contrast, while TFV also involves a form of breaking and entering, and the theft of property, the targets differ and it is typically completed on the street, where the risk of being sighted during the commission of an offense is potentially higher. While the opportunity surface for TFV may overlap with that for residential burglary, differences will also exist, as locations such as car parks offer an abundance of suitable targets combined with periods of uninterrupted access (Briggs, 1991; Kazmierow et al., 2009; Light, Nee, & Ingham, 1993). In addition, recent research (Johnson, Summers, & Pease, 2009) suggests that those who commit residential burglary in an area tend to be different offenders to those who commit TFV. Thus, while on the face of it, residential burglary and TFV may appear to be similar crimes, they are quite different in terms of the spatial distribution of opportunity, how they are committed, and who commits these offenses. Studying patterns of different types of crime is important for their understanding, but also for testing the generality of theories of crime pattern formation. In the present study, we therefore focus our attention on TFV, it being an understudied but common form of crime, and consider how features of the urban environment might affect offender spatial decision making for this type of crime.

### Theories of Environmental Criminology

Routine activity theory (Cohen & Felson, 1979) considers how the availability, movement, and activities of people influence the likelihood of crime occurrence. In particular, the theory states that for a crime to occur, a motivated offender must converge in space and time with a suitable target and must do so in the absence of a capable guardian. From this perspective, crime occurrence is seen as parasitic, with people’s routine activity patterns shaping the opportunities for the necessary conditions for crime to occur.

Crime pattern theory (CPT; Brantingham & Brantingham, 1993) more explicitly considers how people’s routine activities influence their awareness of criminal opportunities, and how this might lead to spatial concentrations of crime. According to the theory, people form mental maps of their routine activity spaces and these are represented as a set of topological features. Routine activity nodes represent the places at which people regularly spend time, and would include a person’s home, place of work, the recreation facilities they visit, and so on. While many routine activity nodes will be idiosyncratic, some will be shared by many people. For example, much of the population will be aware of, and spend time at landmarks such as retail or city centers, and transport hubs (Bernasco & Block, 2009).

Awareness spaces are believed to develop for these nodes of activity and the surrounding areas, and it is where these awareness spaces intersect with suitable opportunities for crime that offenders are most expected to engage in crime. Studies of the journey to crime provide support for this by showing that most offenders commit crime close to their home location (e.g., Rossmo, 2000; Townsley & Sidebottom, 2010), despite the many and varied opportunities available to them (see Reppetto, 1974). In terms of spatial hotspot formation, it is where the awareness spaces of numerous offenders overlap with suitable opportunities for crime that hotspots are predicted to most likely occur.

On the basis of CPT, we would predict that offenders who commit TFV will be more likely to choose locations that are close to their place of residence, city centers, and other routine activity nodes. However, while awareness spaces may be relatively stable on short time scales such as a year or two, they may vary over longer intervals (e.g., Bernasco, 2010), or as a consequence of salient transitions in a person’s life. The transition from childhood to adulthood is likely to be important in this respect. For example, this transition opens up access to alternative means of transportation, which reduces constraints to mobility (e.g., Snook et al., 2005; Townsley & Sidebottom, 2010; see also, Department for Transport, 2013). Moreover, during these two different parts of the life-course, activity nodes such as schools (Baudains, Braithwaite, & Johnson, 2013) and places of work are likely to vary in terms of their relevance, or the way in which they affect people’s routine activities.

Consequently, our expectation is that younger offenders will tend to commit offenses closer to their home location than their older counterparts (Hypothesis 1), and closer to routine activity nodes that are of particular relevance to them. While no studies have examined this for the crime of TFV, previous studies of residential burglary (Clare, Fernández, & Morgan, 2009) and riots (Baudains et al., 2013) demonstrate that younger offenders tend to commit offenses closer to home than do adults. With respect to routine activity nodes, for younger offenders we expect schools to strongly feature in their awareness spaces and hence influence the locations they target (Hypothesis 2a). For adult offenders, we expect schools to have less of an influence on their awareness of criminal opportunities (Hypothesis 2b). In support of Hypothesis 2, studies of offense locations have shown that the risk of crime tends to be higher in the areas that surround schools (e.g., Roncek, 2000). However, as such studies do not consider the characteristics of the offenders involved, it is unclear whether the presence of schools affects all offenders equally. The same is true for recent studies that have used the discrete choice approach to examine the influence of schools on offender decision making (Bernasco & Block, 2009; Bernasco, Block, & Ruiter, 2013; Bernasco et al., 2012), as these studies do not usually examine whether observed effects differ for young and older adults (for an exception, see Baudains et al., 2013).

In the United Kingdom, city centers are an important landmark for most people as they contain, among other things, major shopping developments.^1^ Consequently, we predict that the probability that offenders will commit crimes in an area will increase the closer that area is to the city center (Hypothesis 3a). However, given that older and younger adult offenders appear to differ in terms of their mobility (e.g., Clare et al., 2009), unless an offender lives in or adjacent to the city center, it seems likely that ceteris paribus this routine activity node will have a greater influence on older offenders than their younger counterparts (Hypothesis 3b).

Public transportation is popular in the United Kingdom and hence mass transit facilities, such as train stations, are consequently likely to represent a routine activity node for many people. For this reason, we predict that, all else equal, offenders will be more likely to commit offenses in an area if that area contains a train station (Hypothesis 4a). However, rail usage in the United Kingdom varies by age (e.g., Department for Transport, 2013) such that those above the age of 18 are more likely to use this form of transport. Thus, train stations are more likely to represent routine activity nodes for adult offenders, and consequently we expect their influence to be particularly prominent for older offenders (Hypothesis 4b).

As articulated above, people’s awareness spaces are likely to be influenced by constraints to mobility, and as such distance can be considered a measure of impedance that influences the likelihood that they will become familiar with a particular location. However, people’s awareness of locations is affected by things other than distance. For example, while the distance between two places provides one indication of how easy or likely it is that people may travel between them, two pairs of locations that are the same Euclidian distance apart may differ substantially with respect to how easy it is to actually travel between them.

On the basis of CPT, a logical prediction is that places that are easily accessible—as determined by the configuration of the street network—should experience more crime than those that are not. However, increased usage also provides a supply of potential guardians against crime (Jacobs, 1961), meaning that crime could instead be suppressed at such locations. Much of the research (for exceptions, see Hakim, Rengert, & Shachmurove, 2001; Hillier, 2004; Hillier & Shu, 2000) concerned with the connectivity of places, where places have been defined in terms of neighborhoods (White, 1990), street segments (Beavon, Brantingham, & Brantingham, 1994; Bevis & Nutter, 1977; Johnson & Bowers, 2010), or individual homes (Armitage, 2007), suggests that the risk of burglary (at least) is higher at those places that are more connected to others (see also, Taylor & Gottfredson, 1986). However, such studies are based on the analysis of where crimes occur, without reference to where offenders live. Thus, one problem with such studies is that any inference regarding how offender awareness spaces might influence their targeting choices is clearly indirect. For instance, it is possible that the findings discussed so far reflect systematic variation in omitted variables, such as the distribution of offender residences. Without considering both where offenders live *and* where they offend, it is not possible to rule out such a possibility.

To our knowledge, to date no study has attempted to measure the effect of street network accessibility on offender spatial decision making while controlling for the location of offender residences. However, one study (Clare et al., 2009) has examined how spatial distributions of residential burglary could be jointly influenced by the location of offender homes and the accessibility of the area as determined by the *train network*. The results of that study suggested that there was an increased probability of an offender committing offenses in an area if that area, and the one within which the offender resided, had a train line running through them. However, the role that the street network might play in connecting locations, and hence shape offender spatial decision making, was not explored in that study.

As the street network is routinely used by people (offenders or otherwise) to move from one place to another, it is reasonable to suggest that this type of connector should substantially influence the targeting choices of offenders, and for this reason we examine this issue here, and do so more directly than has been the case hitherto. On the basis of CPT, we expect that offenders will be more likely to target a location if that location is connected to others via the network of major roads (Hypothesis 5a). Moreover, as discussed above, we anticipate the mobility of adult offenders to be more extensive than that of their younger counterparts (e.g., Snook et al., 2005) and hence to be more strongly influenced by the network of major roads. The principle role of such roads is, after all, to facilitate travel between rather than within areas. Consequently, we predict that the role of connectivity will be particularly important for older offenders (Hypothesis 5b). Of course, other factors will affect offender spatial decision making, and in the next section, we consider a different theoretical perspective.

### Social Disorganization Theory

Theories of social disorganization focus on how the social composition of a neighborhood may make it resistant (or otherwise) to criminal activity (e.g., Bursik, 1988; Shaw & McKay, 1942). Central to such theories is the concept of social cohesion (Sampson, Raudenbush, & Earls, 1997) that, where it exists, allows residents to act collectively to deter crime. Scholars have argued that social cohesion is more likely to emerge in neighborhoods with stable populations that provide more opportunities for social ties to form (e.g., Coleman, 1988), and in homogeneous communities where residents are more likely to share similar goals and beliefs. Ethnic diversity is a factor that has, in particular, been discussed as a barrier to social cohesion (e.g., Sampson & Groves, 1989), but variation in other characteristics of the residential population may affect the likelihood that they will be cohesive. For instance, neighborhoods may be less cohesive where their residents are from very different socioeconomic backgrounds (see Hirschfield & Bowers, 1997).

In terms of the mechanisms through which social disorganization might influence the likelihood of crime occurring in a neighborhood, there are at least two possibilities. First, residents may exert informal control over others who live in the neighborhood, reducing the probability that they will engage in crime. Second, for offenders who have decided to commit an offense, their perceptions of social cohesion may influence *where* they decide to do so. With respect to the latter, Bernasco and Nieuwbeerta (2005) suggest that social cohesion may act as a form of impedance that deters offenders from targeting a neighborhood. From this perspective, we predict that offenders will be more likely to target areas in which the composition of residents is not conducive to the formation of a cohesive community (Hypothesis 5). This hypothesis receives support from previous studies of residential burglary (Bernasco & Nieuwbeerta, 2005; Clare et al., 2009) but has not been tested for TFV.

In the next section, we discuss the analytic strategy to hypothesis testing adopted. We then consider the data acquired for analysis and its provenance. In the subsequent section we present the results, and conclude by discussing the implications of the findings for theory and crime reduction policy.

## Analytic Strategy

Research studies concerned with the spatial distribution of crime usually examine either where crimes are committed or where offenders live. The former usually assume that places will systematically differ in the extent to which they are conducive to crime, and the aim of analysis is to test attempts at explanation. The latter studies typically focus on how the composition of an area might instead contribute to the number of motivated offenders residing within it. Because each of these set of studies fail to control for the variables examined by the other, interpretation of the findings can be difficult, and even confounded. For example, situations may arise whereby otherwise vulnerable areas may be observed to experience little or no crime if no offenders live near to, or are aware of them. Likewise, areas that are otherwise not conducive to crime might experience considerable volumes of crime simply because many offenders live nearby. Therefore, to really enhance understanding of spatial crime patterns, ideally both the characteristics of the area in which offences occur and those in which offenders reside should be studied jointly.

Simultaneously modeling where offenders reside and where they offend requires a different approach to analysis than is typically used. Bernasco and Nieuwbeerta (2005) re-imagine the approach to studying offender spatial decision making by using a discrete choice approach (McFadden, 1973). In their study, they examined how the areas that offenders committed offenses in differed from those that they could have targeted, but did not. Using such a framework enabled them to estimate, within the same model, the influence of the characteristics of the potential target areas and the proximity of the offenders’ homes on offender spatial choices. In contrast to other studies that consider only the distance between where offenders live and where they offend (e.g., Wiles & Costello, 2000), the discrete choice approach uses distance as just one of a set of *independent* variables—the choice of area in which offenders commit offenses being the dependent variable.

The type of model used, also referred to as a random utility model (for an overview, see Train, 2003), assumes that when making a decision, a chooser (in this case an offender) selects from a set of alternatives the choice that maximizes their perceived utility. What the set of alternatives is can vary according to the theory under investigation. In the case of offender target selection, it has been suggested that offender target selection is a multi-stage process, whereby offenders first select an area within which to offend and then select a specific target (e.g., Cornish & Clarke, 1986). With this in mind, Bernasco and Nieuwbeerta (2005) focus on the first stage of this process, and examine those factors that differentiate the areas where residential burglars do and do not commit offenses.

A number of studies have subsequently used this approach to analysis for a range of crime types. For instance, Clare et al. (2009) examined the spatial decision making of residential burglars in Perth, Australia; Bernasco and Block (2009) examined the spatial decision making of street robbers in Chicago, Illinois, and Baudains et al. (2013) recently examined the spatial decision making of rioters in London, UK. In all cases, the study authors examined how offender decision making was influenced by a range of factors, including but not limited to, distance.

An important and desirable feature of the approach is that like other multivariate approaches, the contribution of a particular variable is estimated after controlling for the influence of all others. Thus, for example, after having controlled for the importance of propinquity, one can estimate the potential role of other factors on offender decision making. Moreover, where choosers (offenders) feature in the data multiple times due to repeat offending, the dependency in the data may be dealt with by computing standard errors that account for such clustering (White, 1982).

For the reasons discussed above, we use a discrete choice modeling approach here. To specify the model, for each choice, we denote j as a member of the set of alternatives from which a chooser i must select a single area. In this case, the set of alternatives is given by the set of census Lower Super Output Areas in our study area, Dorset, UK (see below). In line with the rational choice perspective (Cornish & Clarke, 1986), we assume that when faced with the decision of which member of the set of alternatives to offend in, offender i will choose the alternative that maximizes their utility. Put differently, if the utility for offender i choosing zone j in which to offend is given by $U_{ij},$ then offender *i* will choose the area k such that $U_{ik}>U_{ij}$ for all j≠k.

The utility $U_{ij}$ derived by offender i from choosing zone j is modeled as follows:

$$U_{ij}=V_{ij}+\varepsilon_{ij},$$

where $V_{ij}$ is the utility associated with offender i choosing zone j based on some systematic set of preferences over the population (to be estimated). In contrast, $\varepsilon_{ij}$ is the utility gained from unobserved personal preferences and the idiosyncrasies of each offender.

And,

$$V_{ij}=\overset{M}{\underset{m=1}{\sum }}\beta_{m}X_{mij},$$

where M is the number of independent variables for which data are captured at the area level and included in the model. For example, $X_{mij}$ is the measured value of attribute m for offender i choosing to offend in zone j. The $\beta_{m}$ are empirically estimated parameter values associated with each attribute m in the evaluation of the utility of each available choice. Where an attribute $X_{m}$ is estimated to influence observed choices, the associated $\beta_{m}$ will differ significantly from zero.

Assuming that the error terms that account for the idiosyncrasies over the population are independently and identically distributed according to an extreme value Type I distribution (i.e., Gumbel distribution), one can show that the probability an offender chooses zone j is given by (McFadden, 1973):

$$P(Y_{i}=j)=\frac{exp(V_{ij})}{\sum_{k=1}^{J}exp(V_{ik})}=\frac{exp(\beta_{1}X_{1ij}+\beta_{2}X_{2ij}+\ldots +\beta_{M}X_{Mij})}{\sum_{k=1}^{J}exp(\beta_{1}X_{1ik}+\beta_{2}X_{2ik}+\ldots +\beta_{M}X_{Mik})},$$

where j is the number of zones available for the offender to choose between. This is the conditional logit model, and the $\beta_{m}$ may be estimated using maximum likelihood estimation. As the $exp(\beta_{m})$ are partial coefficients, they can be interpreted as the multiplicative effects of a one-unit increase in a particular attribute of an area on the probability of chooser *i* selecting that area. Thus, if an $exp(\beta_{m})$ equals 1, this means that there is no association between variable *m* and offender spatial decision making. Values above (below) one suggest that the odds of an area being chosen is positively (negatively) associated with the variable considered.

In addition to modeling the influence of area-level attributes, it is possible to include interaction terms to allow estimation of the effects of factors that vary systematically across choosers. To illustrate, in the present study, we are interested in how the age group to which an offender belongs influences their spatial decision making (e.g., Hypothesis 1). To do so, for example, instead of modeling distance using a single vector, as would be the case for the simple model:

$$P(Y_{i}=j)=\frac{exp(\beta_{1}D_{ij})}{\sum_{k=1}^{J}exp(\beta_{1}D_{ik})},$$

where *D_ij_* is the distance between the area in which offender *i* resides and area *j*, we include two binary variables to model how the age group to which the offender belongs might influence the distance traveled, as follows:

$$P(Y_{i}=j)=\frac{exp(\beta_{1}A_{i}D_{ij}+\beta_{2}J_{i}D_{ij})}{\sum_{k=1}^{J}exp(\beta_{1}A_{i}D_{ik}+\beta_{2}J_{i}D_{ik})},$$

where attribute *A_i_* is a binary variable coded as 1 if an offender is an adult (aged 18 years or older), and 0 otherwise. Likewise, attribute *J_i_* is a binary variable that is coded as 1 if an offender is a juvenile (aged less than 18 years), and 0 otherwise. In the above example, to examine whether the age group to which an offender belongs interacts with distance we test the null hypothesis that $\beta_{1}=\beta_{2}$. All models are estimated using STATA 10 SE.

## Data

### Police Detection Data

Data for crimes detected by the police were acquired for the county of Dorset (UK) for the 5-year period January 1, 2001, to December 31, 2005. In the United Kingdom, crimes detected by the police include both primary detections, which are solved through investigative effort and, in contrast to many other countries, those that are taken into consideration (TIC). The latter are crimes that an offender will volunteer information about to the police at the time of arrest. If the details supplied by the offender can be verified, and the information supplied by the offender satisfies other legal criteria, the offender will accept those offenses. One incentive for an offender to volunteer such information at the point of arrest is that if they are convicted for a series of offenses at the same time, the sentence for each offense may, under some conditions, run concurrently; an offender cannot be convicted of the same offense more than once, so admitting to these offenses may ultimately be beneficial. The legal validity of such crimes means that detection rates for some crimes may be higher in the United Kingdom than those in other countries. In the present study, the detection rate for TFV was 17%.

For each offense, the available data included the location and date, the offender’s age at the time of the offense, and their place of residence, accurate to a resolution of 1 m. Only those offenses where geographical coordinates were available for both the offense and offender, home locations were analyzed. For some offenses (24.3%), more than one offender was involved in a crime. Most studies that apply the discrete choice approach exclude such offenses (e.g., Bernasco, 2010; Bernasco & Block, 2009, 2011; Clare et al., 2009) because the approach to modeling assumes a single decision making agent. This leads to considerable attrition in the data that may be problematic here, as older and younger offenders may engage in co-offending to differing degrees. For this reason, where more than one offender was involved in an offense, we adopt the approach taken by Bernasco et al. (2013) and randomly select the data for one offender for each offense. As with Bernasco et al. (2013), we repeated the analysis for offenses that involved only single offenders. However, the results were identical, and so we discuss them no further.

In line with previous studies (Bernasco, 2006, 2010; Bernasco & Block, 2009, 2011; Bernasco & Luykx, 2003; Bernasco et al., 2013; Clare et al., 2009), crimes committed by offenders living outside the study area were excluded from the sample. This is a limitation of the method that applies to all studies of this kind but it is a necessary one. The reason is that the approach to analysis requires that all alternatives in the choice set (areas that are and are not selected) be enumerated for the purposes of parameter estimation. Including all possible choices outside of the study region would require more data (potentially all areas in the United Kingdom) than were available for analysis.

A total of 721 crimes committed by 263 offenders were included in the analyses. The dependency in the data associated with reoffending has the potential to lead to errors of statistical inference. Specifically, if they are treated as independent choices, the decisions of prolific offenders can disproportionately influence parameter estimation and lead to downward bias in the standard errors. For this reason, as in previous work (e.g., Bernasco & Nieuwbeerta, 2005), robust standard errors (*SE*)—clustered by offender—were used to account for the nested structure of the data.

**Figure 1.**
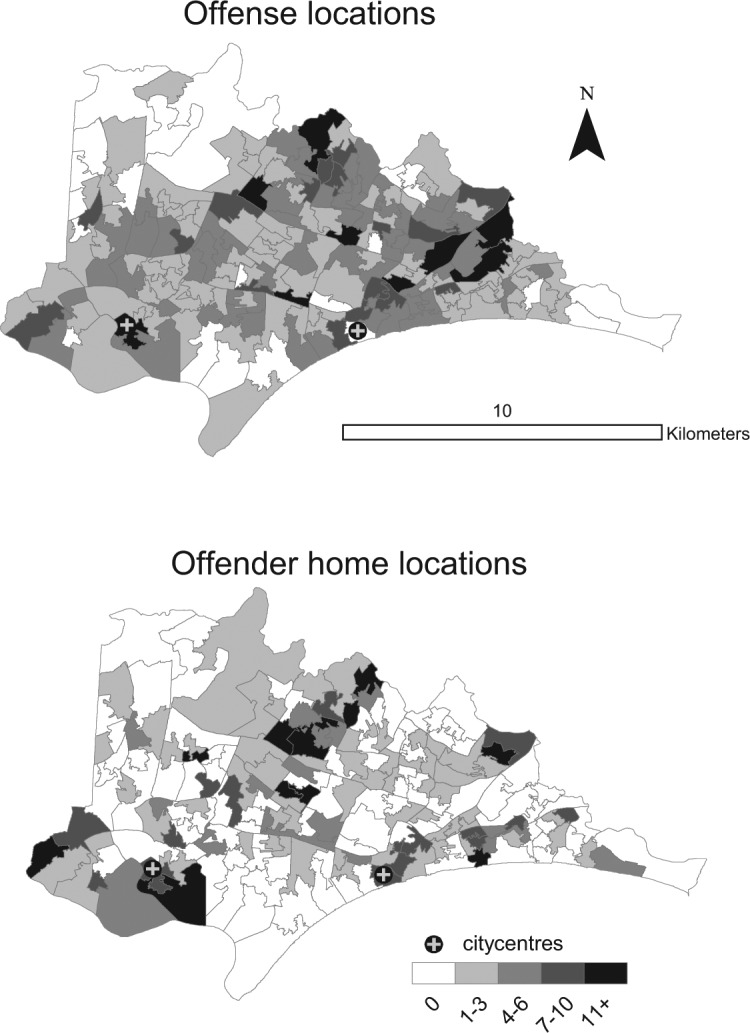
Thematic map of theft from vehicle (TFV) aggregated offense and offender home locations for the U.K. Census Lower Super Output Areas in the study area.

The unit of analysis selected was the U.K. census Lower Super Output Area (LSOA). For the study area, there were 198 LSOAs, each with a population of around 1,524 people and about 662 residential households. LSOAs are somewhat smaller than the areas used in most (but not all; for example, Bernasco et al., 2013) previous studies of this kind. For example, in the Bernasco and Nieuwbeerta (2005) study, the areas selected had an average population of 4,952 and around 2,380 households. Using a Geographical Information System (GIS), the police data were “related” to maps of the area to determine origin-destination flows. For the purposes of illustration, Figure 1 shows a map of the LSOA geography.

### Crime Pattern Theory Variables

Table 1 provides descriptive statistics for the independent variables used. To compute the distances for each of the possible origin-destination flows, we calculated the distance between each centroid and every other. Where the two areas were the same, as would be the case where an offender lived within the LSOA within which they offended, in line with Bernasco and Nieuwbeerta (2005), we estimated the distance that they would have traveled using the Ghosh (1951) correction of $0.5\sqrt{S},$ where *S* is the geographic area of that neighborhood in square kilometers.^2^ To compute the distance of each area from the city center, we computed the distance between that LSOA and the centroids of each of the two main centers of activity within the wider study area (i.e., Bournemouth and Poole city centers; see Figure 1), and used whichever was the shortest.

**Table 1.** Summary Statistics for the Independent Variables at the LSOA Area Level.

	*Mean*	*SD*	Min	Max
Crime pattern theory (CPT)				
Distance to offender’s home (km)	5.66	3.15	0.19	18.98
Distance to city center (km)	3.72	1.60	0.33	8.36
Presence of school(s) (1/0)	0.37	0.48	0.00	1.00
Presence of train station(s) (1/0)	0.03	0.17	0.00	1.00
Connectivity: Major road	0.46	0.50	0.00	1.00
Social cohesion				
Population turnover (10%)	2.47	1.14	1.14	7.67
Socioeconomic heterogeneity (10%)	8.62	0.21	7.13	8.87
Opportunity				
Number of car parks^a^	0.34	0.73	0.00	4.00
Number of cars/vans	783.79	152.20	474.00	1,252.00

*Note*. LSOA = Lower Super Output Area.s

a Three quarters of LSOAs (151) had no car parks. When these are excluded, the mean number of car parks per LSOA is 1.43 (*SD* = 0.85).

Connectivity can be measured in a number of ways. For example, it could be operationalized by examining variation in the extent to which areas have major roads running through them (see White, 1990). Here, we measure it more specifically by determining which combinations of areas are directly connected via the network of major roads.^3^ To do this, Ordnance Survey (OS) MasterMap data were used to identify which areas contained main roads (76% of LSOAs), and a binary variable was coded with a value of one where this was the case, zero otherwise. We then used these data to determine which pairs of areas were connected to each other by the system of major roads (46% of all possible pairs were connected by this network). In the event that two areas considered (i.e., the offender’s home location and each area in the set of alternatives) were connected in this way, when generating the data for analysis, a binary variable was coded with a value of one, zero otherwise. In addition, as connectivity so derived could simply indicate that two areas are next to each other, we also included an adjacency variable so that we could isolate the influence of connectivity after controlling for adjacency.

OS MasterMap data were also used to count the number of schools/colleges (those that would be attended by children up to the age of 18 years) located within each LSOA. For mass transit stations, we identified those areas in which there was a rail station. There were six in the study area.

### Social Cohesion Variables

Population turnover was calculated by computing the number of people who had moved into the area in the previous 12 months, divided by the total population for the area, multiplied by 100. As in Bernasco & Nieuwbeerta’s (2005) study, we divide these estimates by a factor of 10. This scaling has no effect on the statistical significance of the parameter estimates and merely serves to make the parameters easier to understand.

There was little variation in ethnicity across the study area and for this reason, we did not attempt to estimate how ethnic heterogeneity might influence offender spatial decision making. We did, however, examine how heterogeneity in socioeconomic groups might influence offender target choices (Hirschfield & Bowers, 1997). Socioeconomic heterogeneity was calculated using the index of qualitative variation (Agresti & Agresti, 1978; Wilcox, 1973), which is derived in the following way:

$$SE_{j}=(1-\overset{n}{\underset{k=1}{\sum }}p_{kj}^{2})\times 100,$$

where n is the total number of different socioeconomic groups and $p_{kj}$ is the proportion of individuals belonging to socioeconomic group k that reside in zone j. The index can be interpreted as indicating the probability that any two people selected at random from an area will belong to different socioeconomic groups, with larger values indicating more heterogeneity. In this case, the index was derived using data from the 2001 U.K. Census, and indicated the extent to which people in an area belonged to the same socioeconomic groups. The data were classified into six groups: (a) managerial or other professional occupations; (b) intermediate occupations and small employers; (c) lower, (semi-)routine occupations; (d) never worked or long-term unemployed; (e) full-time studies; and (f) other. As with the index of population turnover, to ease interpretation, and for consistency with previous research (e.g., Bernasco & Nieuwbeerta, 2005), we multiplied this heterogeneity coefficient by a factor of 10.

### Control Variables

As two basic indicators of opportunity, we included the number of car parks—derived using U.K. Department for Transport data—and the number of registered cars and vans in an area, estimated using data from the 2001 U.K. Census.

## Results

Table 2 shows the results of the conditional logit model, organized by theoretical perspective. It should be noted that with respect to overall model fit, the pseudo *R*^2^ values associated with the conditional logit model are always much lower than those associated with (for example) ordinary least squares regression models. In fact, McFadden (1979) states that *R*^2^ values above .20 are to be considered an excellent fit to the data (p. 309).

**Table 2.** Odds Ratios $e^{\beta }$ for Each Variable of the Conditional Logit Model (*p*-values shown are one-tailed tests). Note that distance measures shown are logged values.

	$e^{\beta }$	*Z*
Crime pattern theory (CPT)		
Distance to offender’s home (log km)		
Adults	0.18*	−5.20
Juveniles	0.07*	−9.29
Distance to city (log km)		
Adults	0.40**	−2.35
Juveniles	2.49**	2.72
Presence of school(s)		
Adults	1.09	0.85
Juveniles	1.43**	2.81
Presence of train station		
Adults	1.73***	1.83
Juveniles	1.59	1.16
Connectivity—major road(s)		
Adults	1.98**	2.29
Juveniles	1.13	0.34
Adjacency	1.12	0.60
Social cohesion		
Population turnover (10%)	1.20*	3.31
Socioeconomic heterogeneity (10%)	1.73***	1.97
Opportunity		
No. car parks	1.01	0.22
No. cars and vans	1.01***	1.98

*Note*. Wald = 407.21, Log-Likelihood = −3235.20, Pseudo *R*^2^ = 0.15.

* *p* < .001. **p < .01. ***p < .05 (one-tailed).

Considering the CPT variables first, as predicted (Hypothesis 1), there was a significant negative effect of distance, with offenders being more likely to target areas that were closer to where they lived. In Table 2, distance is measured on a logarithmic scale. The rationale for transforming the data in this way is that (for example) every additional 1 km traveled is likely to be perceived as more important for shorter than for longer trips, and using a log transformation accounts for this. Analyses conducted using the untransformed Euclidian distances (for adults, $e^{\beta }=0.73,$ *p* < .001; for the juvenile offenders, $e^{\beta }=0.54,$ *p* < .001) confirmed that the logged values provided a better fit to the data. However, the coefficients associated with the logged distances are a little difficult to interpret as one has to think in terms of logged distances, and so for the purposes of illustration, we consider the coefficients for the untransformed data. In this case, the odds of an adult (juvenile) offender selecting an area decreases by a factor of 0.73 (0.54) for every km that area is located from where they live. As predicted, the difference in the coefficients associated with the logged distances for the younger and older offenders was statistically significant $\chi^{2}=5.88,$ *p* < .01, one-tailed). Descriptive statistics further illustrate the point, indicating that adult offenders (*M* = 2.24, *SD* = 2.63) typically traveled further than did younger offenders (*M* = 0.67, *SD* = 1.57).

In line with Hypothesis 2, the presence of schools appeared to influence the spatial decision making of the younger offenders, for whom they would be potential routine activity nodes. As predicted (Hypothesis 2a), this type of facility had little or no influence on the spatial decision making of adult offenders.

In contrast, the odds of an adult offender targeting an area increased the closer it was to the city center. The opposite pattern was observed for juvenile offenders. This is not entirely inconsistent with Hypothesis 3a, but it is surprising that for the younger offenders there was a trend in favor of them targeting areas that were located further away from the city center, rather than toward it. This could be explained by a variety of factors and we will return to this issue in the discussion section.

As predicted, all else equal, the presence of a train station in an area increased the odds that an offender would commit an offense in that area. However, while the coefficients were positive for both adult and juvenile offenders, only that for adult offenders was statistically significant.

Considering connectivity, as predicted (Hypotheses 5 and 5a), the odds of an adult offender targeting an area increased by a factor of about two, if it was connected to the area in which they lived by the network of major roads. In the case of the juvenile offenders, the estimated effects were in the right direction but not statistically significant. The adjacency variable was in the expected direction, but non-significant.

Turning to our two measures of social cohesion, the findings also provide support for social disorganization theory. All other things being equal, for every 10% increase in population turnover, the odds of an offender targeting such an area increased by a factor of 1.20. Similarly, for every 10% increase in social heterogeneity, the odds of an offender targeting the area almost doubles.

Considering the two control variables, the number of car parks in an area did not appear to influence offender target choice. The number of registered vehicles in an area did, however, have a small but significant effect, with offenders favoring areas with more registered vehicles.

## Discussion

In the present article, we examined offender spatial decision making for a high-volume acquisitive crime that has received little attention in the literature, *TFV*. The aim of so doing was twofold. First, to test theories of spatial decision making for a different type of crime that is committed under different conditions to those examined hitherto, thereby testing the generality of the theoretical perspectives considered. Second, to examine particular expectations suggested by theoretical models that had not been tested so far. Assuming that offender spatial decision making for this type of crime is non-random, according to CPT one would expect offenders to be most likely to target areas that are within their awareness spaces and that are the most accessible. As this type of crime generally occurs outside, where offenders can potentially be seen during the commission of an offense, one would also expect that offender perceptions of risk, such as that associated with social cohesion, might also have a part to play in their selection of areas within which to offend.

Using a discrete choice framework, we find evidence to support both theoretical perspectives. For our sample at least, offenders were more likely to target areas that were close to where they lived, and that were likely to include routine activity nodes of importance to their age group—schools in the case of juveniles, the city center and rail stations in the case of adults. As predicted, given their relatively increased (likely) mobility, although they tended to commit offenses close to their home area, adult offenders were found to travel further distances to commit offenses than their younger counterparts. Their spatial decision making also appears to be influenced by how easy it would be to travel from their home location to potential destinations via the network of major roads, as predicted. That this was not the case for the juvenile offenders warrants further attention. One potential explanation for this finding is that, for our sample at least, the crime trips made by juveniles were simply too short for the road network to play an important part in shaping their target choices.

The influence of the road network on adult offender spatial decision making is a particularly novel result and this finding, along with those concerned with the impact of routine activity nodes on offender spatial decision making, has potential policy implications for those involved in the development of urban spaces. This is the case insofar as the results suggest that the design of environments, in terms of where routine activity nodes are located and how areas are connected, may not only shape opportunities for crime to occur but also which opportunities offenders are most likely to exploit. To elaborate, the findings suggest that all other things being equal, when faced with the choice of which of a set of areas to target, an adult offender is more likely to target those that are more accessible via the street network. The results also suggest that the placement of new facilities that might attract people to an area could increase the probability with which crime will occur in them. Thus, our findings suggest that urban planners involved in the building of new developments, or in extending or making changes to the road network, should consider the potential influence of their decisions on the risk of crime in an area. Conducting a formal crime impact assessment (for a further discussion, see Bowers, 2014; Ekblom, 1997, 2002) that is informed by findings such as those presented here would be one way of doing this.

The fact that younger offenders did not appear to exhibit a preference for targeting areas that were closer to the city center, but appeared to prefer those located further away is a potentially puzzling finding. However, an important point to appreciate when considering this is that the distance from each area to the city center is considered independently of how far *the offender’s home* is from the city center. Therefore, the pattern observed could simply be the result of younger offenders being more likely to commit crime closer to their homes (confirmed by our analyses) and to also live in areas that are further away from the city center. Follow-up analyses provide some support for this by showing that on average younger offenders lived further (4.4 km) from the city center than did their older counterparts (3.8 km). However, this finding deserves further exploration in future research.

In line with social disorganization theory, it appears that offenders were more likely to target those areas in which residents have the least potential to form social ties, either because population turnover is relatively high, or because the residents come from different socioeconomic backgrounds (or both). This result is in line with those of Bernasco and Nieuwbeerta (2005), who find that for residential burglary the social composition of an area may influence offender decision making by deterring offenders from targeting those locations in which social cohesion is most likely. Baudains et al. (2013) have also recently shown—using a discrete choice model—that some proxies for social cohesion (i.e., deprivation and population turnover, but not ethnic diversity) appeared to be associated with offender spatial decision making during the 2011 London riots.

These results also complement previous research that demonstrates the influence of social cohesion and related neighborhood influences in explaining the spatial distribution of crime (e.g., Sampson, 2012; Sampson & Groves, 1989; Sampson, Morenoff, & Gannon-Rowley, 2002; Weisburd, Groff, & Yang, 2012). They do so by providing support for the role of these factors along with other anticipated influences such as propinquity, therefore lending support both to crime pattern and social disorganization theories. To characterize the theories crudely, CPT focuses on how offender mobility and their routine activities shape their awareness of criminal opportunities. Social disorganization on the other hand considers how the social fabric of a community might affect the extent to which residents can and will mobilize to deter or deflect crime. Thus, the two theories essentially consider crime occurrence through a different lens, primarily considering the roles of two different sets of actors. However, as Weisburd et al. (2012) discuss (but see, Braga and Clarke, 2014), the two theories are clearly compatible, and more research that integrates both perspectives thus would be advantageous.

Drawing on CPT, an increasing body of research—as discussed in the introduction—has examined how spatial distributions of crime risk might be affected by the configuration of the urban environment at the micro level, using units of analysis such as the block face. In line with CPT, for example, research has shown that, after controlling for other factors, the risk of burglary is highest on more accessible street segments and those intended for higher volumes of vehicular or pedestrian use.

However, little research has examined how the configuration of the urban environment might affect the potential for collective action in neighborhoods with varying levels of social cohesion, and this would seem to be a fruitful avenue for research. Considering the mechanisms through which social cohesion might influence the likelihood of crime occurrence, it may do so either through residents influencing the behavior of others in their neighborhood, or by deterring offenders from targeting a particular neighborhood.

Considering the latter, it is possible that collective action will be less effective on particular types of streets, such as major roads that large numbers of non-residents may routinely use for legitimate purposes, and hence on which potential offenders will be more difficult to identify. This could affect the ability of residents to mobilize themselves to deter crime, or offenders may simply perceive that this is the case. Recent studies have begun to explore these issues (e.g., Reynald, 2011; Weisburd et al., 2012), but more research is needed.

In addition to examining variation across places, research might also examine if and how the potential for collective action might vary over time. In the research conducted so far, the ability of a neighborhood to act collectively to deter crime tends to be discussed as if it were relatively time-stable, at least on the time scale of a year or so. However, it is possible that it is much more dynamic, particularly over the course of the day. To elaborate, people’s routine activities vary throughout the day, thereby affecting their potential to act as guardians against crime in the vicinity of their home (see Braga and Clarke, 2014; Johnson and Bowers, 2013). People do, of course, sleep for a good proportion of the day and this will affect their ability to act collectively, but even while they are awake there are constraints to their behavior. Levels of visibility vary throughout the day and this may influence the extent to which residents are likely to, or can monitor, their neighborhood. Thus, the potential for residents to act (collectively) as guardians against crime may systematically vary over the course of the day, being lower during the hours of darkness, for instance.

If this is the case (or for other reasons), offender spatial decision making might also vary by time of day. Testing such hypotheses was beyond the scope of the current study as insufficient data were available to do so reliably. However, future research that does this would make a valuable contribution to the literature.

Of course, as with all studies, a number of caveats are important to discuss. First, social cohesion was estimated indirectly using census data collected for relatively large areal units, as is often the case for empirical studies of this kind (e.g., Bernasco & Nieuwbeerta, 2005; Shaw & McKay, 1942). However, this is rather different to using methods of social observation (e.g., Sampson & Groves, 1989; Sampson et al., 1997) that may be conducted for much smaller spatial units. The use of the latter will undoubtedly be necessary if hypotheses such as those articulated above are to be tested in the future.

Second, it is perhaps surprising that the control variables used here—the number of vehicles in an area and the number of car parks—appeared to have little or no influence on offender spatial decision making. In both cases, the coefficients were in the right direction but they were small and the estimate for the influence of the number of car parks in an area was not statistically significant. This may suggest that, all other things being equal, the abundance of potential targets is not a major influence on the spatial decision making of offenders for this type of crime. However, it is important to consider that the census data concerning the number of vehicles in an area represent the number of *registered vehicles*, rather than the typical abundance of vehicles on the street, and the two may differ considerably, particularly during the day. The number of car parks is less problematic in this sense, but this variable is a crude indicator that simply represents the number of car parks in an area, rather than the number of actual parking spaces available (or used)—data that were unavailable.

Finally, while we examined the influence on offender spatial target choice of some of the routine activity nodes of which offenders might be collectively aware (i.e., city centers, rail stations, and schools), we considered the role of only one that was specific to offenders—the home location. This is typical for studies of this kind as it is usually the only one that is available for analysis. However, using simpler approaches to analysis, researchers (e.g., Wiles and Costello, 2000) have provided evidence to suggest that other idiosyncratic routine activity nodes, such as the home locations of friends, influence offender spatial targeting decisions. Future research using the discrete choice approach might systematically examine the role of routine activity nodes other than the home location, although this will require the analysis or collection of data other than that recorded by the police.

To summarize, using a discrete choice approach, the present study provides further evidence to support crime pattern and social disorganization theory in explaining offender spatial decision making. The present study demonstrates that this is true for a type of crime not studied previously, and for a geographical location not studied elsewhere, thereby extending the external validity of the research base and demonstrating the generality of these theories at explaining the distribution of different types of crime. In addition, we extend criminological understanding by showing that, in line with the expectations articulated in the introduction, the connectivity of an area, and the distribution of routine activity nodes, appear to have a direct effect on offender spatial decision making, and that these effects differ for younger and older offenders.

## Notes

1. Shopping malls, like those found in the United States, are uncommon in U.K. cities, and where they exist, they are often to be found in city centers.

2. A total of 55 offenses occurred in the offender’s home neighborhood. Using the exact distances does not affect model outcomes.

3. There are no motorways in the study area.
